# Toxicity Weighting for Human Biomonitoring Mixture Risk Assessment: A Proof of Concept

**DOI:** 10.3390/toxics11050408

**Published:** 2023-04-26

**Authors:** Miranda M. Loh, Phillipp Schmidt, Yvette Christopher de Vries, Nina Vogel, Marike Kolossa-Gehring, Jelle Vlaanderen, Erik Lebret, Mirjam Luijten

**Affiliations:** 1 Institute of Occupational Medicine—IOM, Edinburgh EH14 4AP, UK; 2 German Environment Agency (UBA), 14195 Berlin, Germany; 3 Institute for Risk Assessment Sciences (IRAS), Utrecht University, 3584 CM Utrecht, The Netherlands; 4 Center for Sustainability, Environment and Health, National Institute for Public Health and the Environment (RIVM), 3720 BA Bilthoven, The Netherlands; 5 Center for Health Protection, National Institute for Public Health and the Environment (RIVM), 3720 BA Bilthoven, The Netherlands

**Keywords:** human biomonitoring (HBM), chemical mixtures, mixture risk assessment, toxicity weighting, health-based guidance value (HBGV), hazard quotient (HQ), hazard index (HI), biomonitoring equivalent (BE), HBM4EU

## Abstract

Chemical mixture risk assessment has, in the past, primarily focused on exposures quantified in the external environment. Assessing health risks using human biomonitoring (HBM) data provides information on the internal concentration, from which a dose can be derived, of chemicals to which human populations are exposed. This study describes a proof of concept for conducting mixture risk assessment with HBM data, using the population-representative German Environmental Survey (GerES) V as a case study. We first attempted to identify groups of correlated biomarkers (also known as ‘communities’, reflecting co-occurrence patterns of chemicals) using a network analysis approach (*n* = 515 individuals) on 51 chemical substances in urine. The underlying question is whether the combined body burden of multiple chemicals is of potential health concern. If so, subsequent questions are which chemicals and which co-occurrence patterns are driving the potential health risks. To address this, a biomonitoring hazard index was developed by summing over hazard quotients, where each biomarker concentration was weighted (divided) by the associated HBM health-based guidance value (HBM-HBGV, HBM value or equivalent). Altogether, for 17 out of the 51 substances, health-based guidance values were available. If the hazard index was higher than 1, then the community was considered of potential health concern and should be evaluated further. Overall, seven communities were identified in the GerES V data. Of the five mixture communities where a hazard index was calculated, the highest hazard community contained N-Acetyl-S-(2-carbamoyl-ethyl)cysteine (AAMA), but this was the only biomarker for which a guidance value was available. Of the other four communities, one included the phthalate metabolites mono-isobutyl phthalate (MiBP) and mono-n-butyl phthalate (MnBP) with high hazard quotients, which led to hazard indices that exceed the value of one in 5.8% of the participants included in the GerES V study. This biological index method can put forward communities of co-occurrence patterns of chemicals on a population level that need further assessment in toxicology or health effects studies. Future mixture risk assessment using HBM data will benefit from additional HBM health-based guidance values based on population studies. Additionally, accounting for different biomonitoring matrices would provide a wider range of exposures. Future hazard index analyses could also take a common mode of action approach, rather than the more agnostic and non-specific approach we have taken in this proof of concept.

## 1. Introduction

The issue of combined exposures to multiple chemicals in humans and the mixture risk assessment and management of those multiple chemicals have received increasing interest in the past decades from researchers, policy makers and concerned citizens alike. Clearly, the human population is exposed to chemical mixtures every day. These exposures originate from a variety of different sources, and exposures occur through diverse pathways—the environment, occupation, diet, use of consumer products, cosmetics, medication and medical implants, and lifestyle factors (e.g., smoking, recreational drugs, tattoo ink). The resulting exposures are complex as is the risk assessment of these chemical mixtures [1,2,3].

To move beyond conducting risk assessments ‘chemical-by-chemical’, the European Commission expressed the ambition to account for the cocktail effect of chemicals when assessing risks from chemicals, with the overall aim to work towards a zero-pollution environment [4]. Among others, the Commission aims to introduce or reinforce provisions to take account of the combination effects in relevant legislations, such as legislation on water, food additives, toys, food contact materials, detergents and cosmetics. For REACH, it will be assessed how to best introduce a mixture assessment factor (MAF) for the chemical safety assessment of substances. EFSA published a guidance providing methodologies for applying scientific criteria and prioritization methods to group chemicals for human risk assessment of combined exposures to multiple chemicals [2]. More recently, EFSA published a roadmap for action on this topic, i.e., the roadmap for action on Risk Assessment of Combined Exposure to Multiple Chemicals (RACEMiC) [1]. Moreover, EC’s Horizon 2020 Research Programme initiated several chemical-mixtures-related research projects [5].

Despite the increased attention for chemical mixtures, still little effort has been devoted to assessing the real-life mixture exposures in the (European) population, thus considerably hampering the mixture risk assessment. HBM4EU, a European Joint Programme funded under Horizon 2020, addressed real-life chemical mixtures exposures in the context of human biomonitoring (HBM) [6]. Chemical mixtures (in the context of HBM) here refer to the common occurrence at the level of the individual of chemical xenobiotic substances measured in blood, urine or other human materials. The overall aim of the work on chemical mixtures in HBM4EU was to improve the precision and efficacy of HBM to inform science, policy and regulatory actions with respect to dealing with mixtures. More specific objectives were:

1. To develop summary indicators to describe the exposure and body burdens of mixtures with an emphasis on defining priority mixtures and drivers of mixture toxicity.
2. To re-evaluate the existing HBM mixture data and collect new HBM mixture data, to identify real-life exposure patterns to mixtures.
3. To further develop and apply practical approaches to identification and assessment of the potential health risks and impacts of mixtures.
4. To inform policy makers, stakeholders and the public at large about mixture exposures and associated health risks.

In this manuscript, we focus on the second and third objective. Our study provides a proof of concept for prioritizing real-life chemical mixtures to which a population is exposed by applying the hazard index (HI) methodology on mixtures identified in HBM data as a first-tier approach to risk assessment of combined exposure to multiple chemicals (further referred to as ‘mixture risk assessment’). For this, we performed a network analysis of data from the population-representative German Environmental Survey (GerES) V held from 2014 to 2017 [7], with the aim to identify patterns in the co-occurrence of chemicals in HBM data. The underlying question is firstly whether the combined body burden of multiple chemicals as measured through HBM is of potential health concern. If so, subsequent questions include which chemicals and which co-occurrence patterns are driving the potential health risks, i.e., have the highest contribution to the hazard index. This could help prioritize mixtures for future risk management to reduce the health risks associated with exposure to these chemicals’ combinations. Here, the network analysis proves useful to delineate patterns of co-occurrence in HBM data. Network analysis is a data-driven approach that identifies dependencies between biomarkers of exposure (for the parent chemical and/or metabolite(s) thereof) measured in the same individual at the same point in time [8]. Recent application of network analysis on multiple datasets from different HBM surveys across Europe [9] confirmed the various opportunities this analysis has to offer with regard to mixture risk assessment. Firstly, network analysis allows for the identification of groups of exposure biomarkers (communities) that are more closely related than others, i.e., the real-life mixtures. Communities give an indication of, on a population level, which biomarkers have similar concentration patterns. For example, Ottenbros et al. found that a number of polychlorinated bisphenyl (PCB) compounds tended to cluster together in a network analysis of the Flemish Environment and Health Survey cord blood samples [8]. Such patterns might be explained by similar exposure patterns or similarity in toxicokinetics, and would be cause for concern in risk assessment when characterized by more than additive toxicity. Secondly, it allows for the identification of determinants that may explain observed variation of patterns in exposure biomarkers. Furthermore, it allows for ranking of individuals based on their cumulative burden of exposure, making the network analysis of HBM data a highly valuable tool for mixture risk assessment.

Mixture risk assessment is complex and typically very demanding in terms of resources and time. Hence, insight into which of the real-life mixtures is of higher concern would be highly valuable. Therefore, in this study, the co-occurrence patterns in GerES V identified through network analysis were weighted using HBM health-based guidance values (HBM-HBGVs) or equivalents developed and/or collected under the HBM4EU project. This resulted in a ‘biomonitoring hazard index’, which greatly facilitates the identification of chemical mixtures that need to be prioritized for human health risk assessment. This paper describes the methodologies applied, presents and discusses the results obtained and reflects on the potential application of the biomonitoring hazard index approach for mixture risk assessment.

## 2. Materials and Methods

*Study data:* The German Environmental Survey for children and adolescents 2014–2017 (GerES V) is a population-representative cross-sectional study carried out to determine the exposure of the general population in Germany to pollutants and their sources. GerES V investigated children and adolescents by determining, on a representative basis, the body burden of environmental pollutants and the exposure to pollutants at home. It was performed in a stratified randomly selected sample consisting of 2294 children and adolescents aged 3 to 17 years and living in 167 different sampling locations in Germany [7,10].

In GerES V, different substances were measured in subsets of participants. Although GerEs V included both blood and urine sampling, these were not collected at the same time. Rather, they were collected approximately six months apart, on average. We therefore chose to base our analysis on one matrix. To avoid high proportions of missing data, urinary biomarkers available for all participants and additional urinary biomarkers available in a subgroup of GerES V participants (*n* = 515) aged 3 to 17 years were included in this analysis. All urinary biomarkers were measured in first-morning void urine samples.

The following HBM4EU priority substances were available in first-morning void urine samples in the subsample of 515 participants: cadmium (Cd), chromium (Cr), mercury (Hg), phthalates, Di-isononyl cyclohexane-1,2-dicarboxylate (DINCH), bisphenol A, polyaromatic hydrocarbons (PAHs), acrylamide, pesticides, aprotic solvents (n-ethyl-pyrrolidone; n-methyl-pyrrolidone), UV-filters (benzophenones (BP)). In addition, the following non-HBM4EU substances were included: antimony (Sb), selenium (Se), parabens, lysmeral and CIT/MIT (methylchloroisothiazolinone/methylisothiazolinone). The methods of analysis for all biomarkers can be found in [11].

Eleven biomarkers were excluded from the network analyses because over 40% of the measurements were below the Limit of Quantification (LOQ). These include phthalate metabolites mono-n-octyl phthalate (MnOP), mono-n-pentyl phthalate (MnPeP), mono-cyclohexyl phthalate (MCHP), mono(propyl-6-hydroxyheptyl) phthalate (OH-MPHP), mono-(2-propyl-6-carboxyhexyl) phthalate (cx-MPHP); aprotic solvents (N-Ethyl-2 pyrrolidone, NEP) metabolite 5-hydroxy-N-ethyl-2-pyrrolidone (5-HNEP), the pesticide glyphosate and its metabolite aminomethylphosphonic acid (AMPA) and the UV-filter metabolites of benzophenone (BP-1 and BP-3). As a result, a total of 51 substances were included in the network analyses (see Table S1).

*Establishing a database of HBM-HBGVs:* For toxicity weighting, we first compiled a database of HBM-HBGVs and equivalents, first focusing on HBM4EU priority substances identified by the HBM4EU chemicals prioritization strategy [12]. Various HBM-HBGVs derived under the HBM4EU project have been derived and published [13,14,15,16,17]. Subsequent focus was on the subset of 51 biomarkers (Table S1) that were further prioritized for the study presented here (see above).

For these 51 biomarkers a scoping review was conducted targeted towards HBM-HBGVs or equivalents, preferably derived by (but not limited to) European or national agencies, organizations or commissions, such as the German Human Biomonitoring Commission [18], ANSES (the French Agency for Food, Environmental and Occupational Health and Safety) or Health and Safety Executive (UK). Guidance values for substances that were not available through agencies in Europe, or that were considered interesting for comparison purposes, were sourced from similar-type organizations outside the region, e.g., US Environmental Protection Agency (US EPA), US Agency for Toxic Substances and Disease Registry (ATSDR) and New Zealand Government—WorkSafe. Descriptions of the different types of guidance values are described in the Supplementary material (Table S2).

*Guidance value selection:* For the substances in the network analyses, HBM-HBGVs were matched on urine, dimension and, if applicable, creatinine standardization. Where appropriate, matching on age-group was performed. For DEHP and DINCH only HBM-HBGVs for the sum of metabolites were available. Two HBM-HBGVs for different DEHP metabolite sums were used per individual, and the higher hazard quotient was chosen. Where no HBM-HBGVs existed, alternative indicators, e.g., Biomonitoring Equivalent (BE), were used.

*Network analysis:* The data preparation steps for the network analysis involved: (a) checking the distribution of the variables; (b) transforming the data if needed; (c) imputation of the data points below the LOD or LOQ (Limit of Detection; Limit of Quantification); (d) correction for outliers; (e) standardization around zero and (f) scaling of the data. Concentrations of biomarkers were natural-log-transformed because HBM distributions are typically skewed. Network analyses were performed as previously described [8,9].

Unweighted network analysis was used to describe the conditional independence between multiple variables, making use of the packages huge and igraph, using R (v3.5.0 or higher) [19,20]. Within these networks, a node or dot represents a biomarker, and an edge or line between two nodes reflects the conditional dependency between these two biomarkers given all other variables.

The graph estimation was conducted using the graphical lasso, which involves penalized maximum likelihood estimation [21]. This method is a simple and fast algorithm for estimation of a sparse inverse covariance matrix using an L1 penalty. The graphical lasso cycles through the variables, fitting a modified lasso regression to each variable in turn. Regularization of the graph was conducted along a sequence of 10 equally spaced lambdas ranging from the maximum lambda (resulting in an empty graph) to the minimum lambda set at 10% of the maximum lambda. Optimal lambda selection was conducted using the stability approach to regularization selection method (StARS), which selects the optimal lambda by variability across subsamples [22]. Variability (or instability) across subsamples is defined as the fraction of times (range: 0–0.5) that two graphs disagree on the presence of an edge, averaged over all edges in the graphs. We used the default variability threshold of 0.1. Within the selected network, the walktrap clustering algorithm from the igraph package was used, which performs random walks (in default of 4 steps) across the network to merge separate communities in a bottom-up manner [23,24,25]. Nodes were colored according to the community they were assigned to; edges linking different communities were colored in red, edges within a community were colored in black.

The actual network analysis was performed on a partial correlation matrix. Therefore, a strategy for dealing with missing data is required. Thus, an (arbitrary) cut-off of a maximum of 40% of HBM levels below the LOD/LOQ (level of detection/level of quantification) was applied. Substances with more than 40% of the measured HBM values below LOD/LOQ were excluded from further analysis. For the included substances, missing values below LOD/LOQ were imputed based on a maximum likelihood estimation via single conditional imputation, dependent on observed values for the other biomarkers. Missing values in biomarkers (completely missing, e.g., due to insufficient sample volume) were imputed by using a single imputation strategy using the R package mice. Observed values were used as prediction matrix for single imputation of the biomarkers (completely missing, e.g., due to insufficient blood volume), using linear regression.

For the network analysis, a correction for creatinine level was performed, to take into account the dilution level of spot or morning urine samples; the dilution level could affect the correlation structure with other substances measured in urine. In addition, we corrected for sex, creatinine, smoking status, age, education, BMI.

*Toxicity weighting approach:* The simplest approach for estimating the combined effect of different components of a chemical mixture is the hazard index (HI) approach, which assumes dose additivity [3]. Note that dose additivity in this first screening exercise was not a requirement for inclusion of a substance in the HI. To derive the HI of a chemical mixture, a Hazard Quotient of each chemical component (HQ_i_) is calculated by scaling the estimated exposure or dose of the component in the population by a level of exposure considered safe or acceptable. For the HI, creatinine-corrected HBM concentrations were used in cases where HBM-HBGVs were creatinine-corrected, and uncorrected values were used where HBM-HBGVs were not creatinine-corrected. The biological hazard quotient for component (HQ_I_) *i* of the mixture is therefore:

$$HQ_{i}\;=\;\frac{\sum_{j=1}^{n}C_{ij}}{HBM-HBGV_{i}}$$

where C_ij_ = the exposure biomarker concentration to component *i* of the mixture for the *jth* individual in the population; *n =* the total population; HBM-HBGV_i_ = the guidance value for the *ith* component of the mixture.

The Hazard Index (HI) of the chemical mixture is then calculated by summing the respective *n* hazard quotients:

$$HI\;=\;\sum_{i=1}^{n}HQ_{i}$$

An HQ or HI value > 1 indicates that exposure to the substance is greater than a threshold level of concern and warrants further investigation.

## 3. Results

### 3.1. Database of HBM-HBGVs

The different types of HBM-HBGVs that were included in the database are described in Table S2. These comprise HBM-GV_GenPop_ and HBM-GV_worker_ that were derived within the HBM4EU project as well as HBM-I and HBM-II values. The former (HBM-GV_GenPop_ HBM-GV_worker_) are equivalent to the HBM-I values from the German Human Biomonitoring Commission [26] and refer to the concentrations in biological media at which no health risk is expected to occur, unlike HBM-II values that represent concentrations above which there is an increased risk for adverse health effects.

Where HBM-HBGVs for a priority substance were not be identified, biomonitoring equivalent (BE) values were obtained from the peer-reviewed literature. BE values are typically derived from pharmacokinetic data to estimate the concentration of a chemical or its metabolite in biological media that is consistent with an existing health-based exposure guidance value (e.g., tolerable daily intake). They are intended for use as screening tools to provide an assessment of which chemical biomarkers are present at levels well below, near, at or above health risk-based exposure concentrations [27].

Workplace guidance values that are concerned with occupational exposures were also obtained; however, these were only considered where HBM-HBGVs and BE guidance values were not available. Given that their derivation considers workplace-specific circumstances with regard to exposure concentrations and patterns, these are considered less relevant for the general population, particularly the children and adolescents which were analyzed here but were used as part of the demonstration of proof-of-concept.

Altogether, HBM-HBGVs were obtained for 20 of the 51 prioritized biomarkers (representing 17 parent substances). For 15 of these, the environmental-based guidance values were obtained, while for two biomarkers (1-hydroxypyrene and total chromium), only occupational HBGVs were available. Multiple HBGVs were identified for most biomarkers (*n* = 16) with most of these being biological equivalents. When multiple HBGVs were available, the lowest value from an authoritative government agency source for the appropriate population was used. We prioritized sources of HBGVs based on our confidence in the comprehensiveness of the review process: HBM4EU, European regulatory agency, other national regulatory agency and finally biological equivalents. Because the latter are conducted without any committee review or agreements, these are likely the most uncertain values. The HBM-HBGVs used for calculating the hazard quotients are shown in Table 1. The information collected on each biomarker of interest along with the type of guidance value can be found in the database of health-based values included in the supplementary material Table S3 (Excel file).

### 3.2. Network Analysis and Toxicity Weighting

#### 3.2.1. Network Analysis

**Figure 1:**
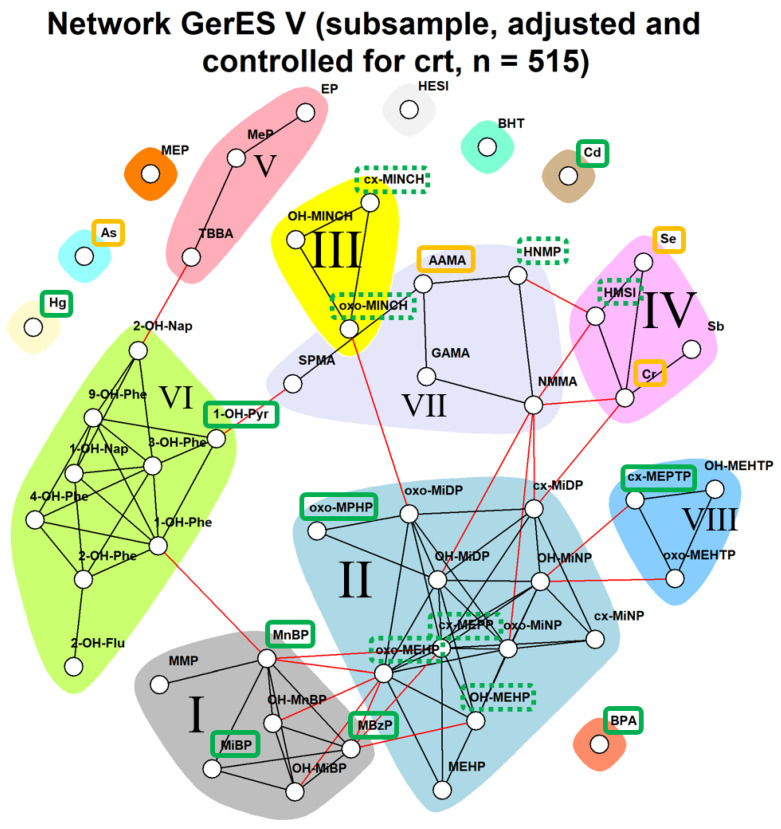
Network and the available guidance values (HBM-HBGVs in green, dashed if the substance is part of a sum; BEs (biomonitoring equivalents) in yellow). Four communities with two or more HBM-HBGVs or equivalents are present. Black lines indicate dependency between nodes (biomarkers) within a community; red lines indicate dependency between nodes in different communities. Chemicals: Cd: cadmium, Cr: total chromium, hg: Mercury (total)), Sb: antimony, Se: Selenium, As: total arsenic, HNMP: (5-hydroxy-N-methyl-2-pyrrolidone), HMSI: (2-hydroxy-N-methylsuccinimide), HESI: (2-hydroxy-N-ethylsuccinimide), AAMA: (N-Acetyl-S-(2-carbamoyl-ethyl)cysteine), GAMA: (N-Acetyl-S-(2-carbamoyl-2-hydroxyethyl)cysteine), TBBA: Tetrabromobisphenol A, NMMA: N-Methylacetamide, OH-MEHTP: (1-mono-(2-ethyl-5-hydroxy-hexyl) benzene-1,4-dicarboxylate), oxo-MEHTP: (1-mono-(2-ethyl-5-oxo-hexyl) benzene-1,4-dicarboxylate), cx-MEPTP: (1-mono-(2-ethyl-5-carboxyl-pentyl) benzene-1,4-dicarboxylate), cx-MINCH: (cyclohexane-1,2-dicarboxylate-mono-(7-carboxylate-4-methyl)heptyl ester), OH-MINCH: (cyclohexane-1,2-dicarboxylate-mono-(7-hydroxy-4-methyl)octyl ester), oxo-MINCH: (cyclohexane-1,2-dicarboxylate-mono-(7-oxo-4-methyl)octyl ester), MEHP (Mono(2-ethylhexyl) phthalate), OH-MEHP: (Mono(2-ethyl-5-hydroxy-hexyl) phthalate), oxo-MEHP: (Mono(2-ethyl-5-oxo-hexyl) phthalate), cx-MEPP: (Mono(2-ethyl-5-carboxy-pentyl) phthalate), MBzP: (Mono-benzyl phthalate), MnBP: (Mono-n-butyl phthalate), OH-MnBP: (3-OH-Mono-n-butyl phthalate), MiBP: (Mono-isobutyl phthalate), OH-MiBP: (2-OH-Mono-iso-butylphthalate), MEP: (Mono-ethyl phthalate), OH-MiNP: (7-OH-(Mono-methyl-octyl) phthalate), oxo-MiNP: (7-Oxo-(Mono-methyl-octyl) phthalate), cx-MiNP: (7-Carboxy-(mono-methyl-heptyl) phthalate), OH-MiDP: (6-OH-Mono-propyl-heptyl phthalate), oxo-MiDP: (6-Oxo-Mono-propyl-heptyl phthalate), cx-MiDP: (Mono(2,7-methyl-7-carboxy-heptyl) phthalate), MMP: (Mono-methyl phthalate), BHT: Butylated hydroxytoluene, SPMA: N-acetyl-S-phenyl-L-cysteine, oxo-MPHP: (6-Oxo-Mono-propyl-heptyl phthalate), oneohnap: 1-NAPH (1-hydroxynaphthalene), twoohnap: 2-NAPH (2-hydroxynaphthalene), twohfluo: 2-FLUO (2-hydroxyfluorene), onehphe: 1-PHEN (1-hydroxyphenanthrene), twohphe: 2-PHEN (2-hydroxyphenanthrene), threehphe: 3-PHEN (3-hydroxyphenanthrene), fourhphe: 4-PHEN (4-hydroxyphenanthrene), ninehphe: 9-PHEN (9-hydroxyphenanthrene), ohpyr: 1-PYR (1-hydroxypyrene), MeP: methylparaben, EP: Ethylparaben, bpa: BPA total (Bisphenol A).

Figure 1 shows the resulting patterns of co-occurring chemicals (depicted in different colors) from the network analysis of the subsample of 515 participants from GerES V when adjusting raw concentrations for creatinine, controlling for creatinine and basic determinants in multivariate analyses in addition. These patterns are referred to as ‘communities’. Several substances were not part of any community, such as some elements (mercury, inorganic arsenic, cadmium), bisphenol A (BPA) and the phthalate MEP. A total of eight communities consisting of three or more substances were observed. These eight communities (mainly) comprised two communities of phthalate metabolites (I, dark grey, and II), DINCH metabolites (III), polycyclic aromatic hydrocarbons (PAHs) (VI), parabens and lysmeral (TBBA) (V), aprotic solvent HNMP, NMMA, acrylamide, its metabolite glycidamide and benzene and its metabolite S-phenyl-mercapturic acid (SPMA) (VII), DEHTP metabolites (VIII), selenium, chromium, antimony and aprotic solvent HMSI (IV). Among the phthalate communities, MMP co-occurred together with BBzP and DnBP and DiBP metabolites (I), while DEHP metabolites co-occurred with propylheptyl phthalate (PHP) and DiNP and DiDP metabolites (II). Thus, communities often reflected the expected co-occurrence of parent chemicals and their metabolites and/or substances from the same chemical family. Additionally, however, also a few communities comprising chemicals from different families were observed.

#### 3.2.2. Toxicity Weighting

**Figure 2:**
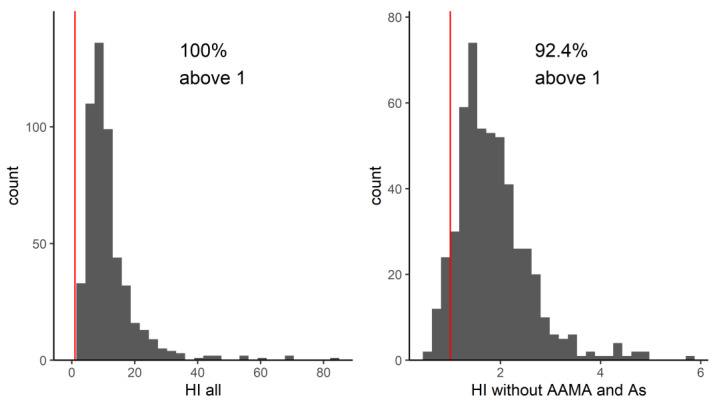
Distribution of hazard indices for all substances with an available guidance value, with and without acrylamide and arsenic. The red line indicates the value of 1.

The first underlying question in this work was whether the combined body burdens of the 51 substances were of potential health concern. A comparison with available HBM-HBGVs or equivalents was possible for less than half of the substances, which led to the calculation of an HI across 20 substances. The resulting HIs (Figure 2) exceed the value of one in 100% of individuals. Thus, with less than half of the measured substances incorporated in the HI, already all subjects had an HI that warrants further investigation. Hence the subsequent question is “which exposures in the HBM data drive the high HI values above 1?” When we look closer at the drivers of the hazard index in the whole population, we see that arsenic and acrylamide are responsible for excessive HI values that decrease by factor 10 when they are removed from the sum (Figure 2). Arsenic was not part of any co-occurrence community. Acrylamide was part of community VII; however, there were no other biomarkers in that community for which a guidance value was available at the time. Therefore, it is not possible to say whether additional biomarkers would have also contributed to the HI for that particular community. 

**Figure 3:**
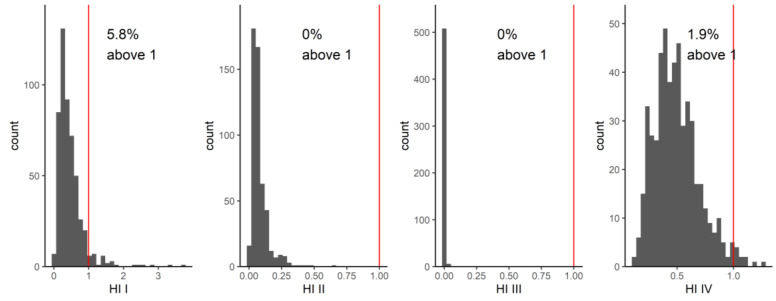
Distribution of hazard indices for communities I to IV. The red line indicates the value of 1. For communities I and IV, a small fraction of the population exceeds an HI value of 1.

**Figure 4:**
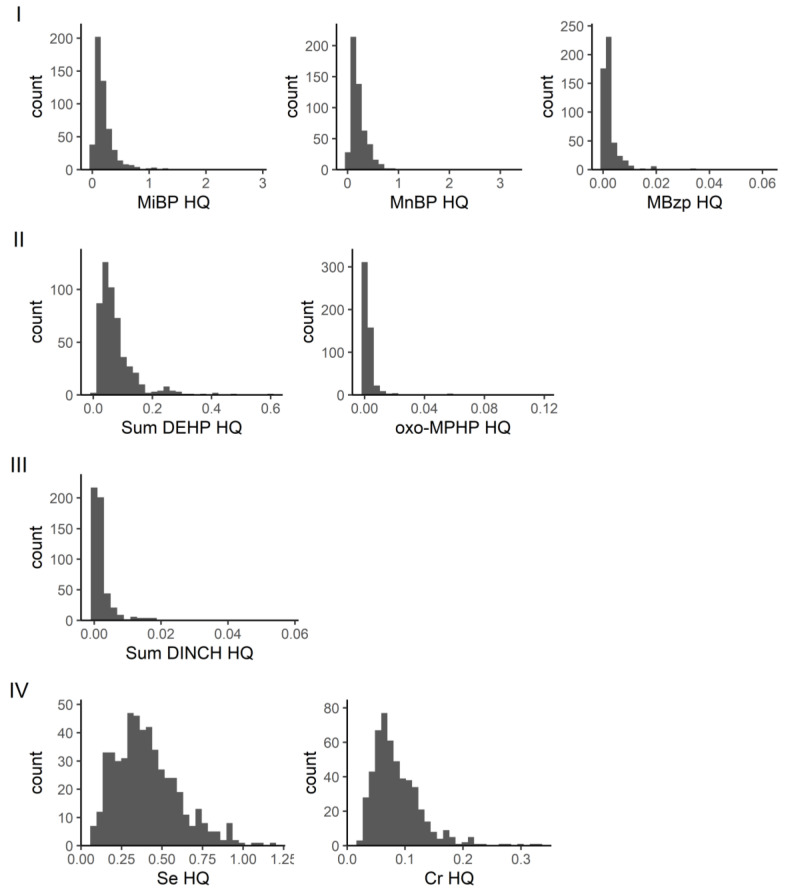
Distribution of individual hazard quotients (HQs) for the substances that are involved in communities I to IV. For DEHP and DINCH, the available HBM-HBGVs for the sums were used (oxo-MEHP, cx-MEPP, OH-MEHP and cx-MINCH, oxo-MINCH, respectively). MiBP (Mono-isobutyl phthalate), MnBP (Mono-n-butyl phthalate), MBzP (Mono-benzyl phthalate), DEHP (Bis(2-ethylhexyl) phthalate), oxo-MPHP (6-Oxo-Mono-propyl-heptyl phthalate), DINCH (1,2-Cyclohexane dicarboxylic acid diisononyl ester).

Of the remaining communities, community I contains the phthalate metabolites MiBP and MnBP with high hazard quotients (see also Figure 4), which leads to HI values that exceed the value of one in 5.8% of the individuals studied (Figure 3). With regard to MiBP (mono-isobutyl phthalate), exceedance of the HBM-HBGVs (resulting in an HQ value > 1) was observed for a small fraction (2.3%, 12 of 515) of the population studied, while one out of 515 exceeded HQ > 1 for MnBP. Community IV comprises, among others, the substances chromium and selenium. As shown in Figure 3, using a BE and a BMGV for calculating the hazard quotients resulted in HI values below one in nearly all participants, i.e., HI values > 1 was observed for 1.9% of the individuals studied. In contrast, the HI values obtained for communities II and III, comprising four phthalate metabolites and two DINCH metabolites, respectively, remained well below the value of 1 (Figure 3). Hence, within the current dataset and via the network analysis, in addition to single chemicals, the communities VII and I would pose the highest concern and thus should be given priority when addressing health risks related to combined exposure to multiple chemicals.

**Figure 5:**
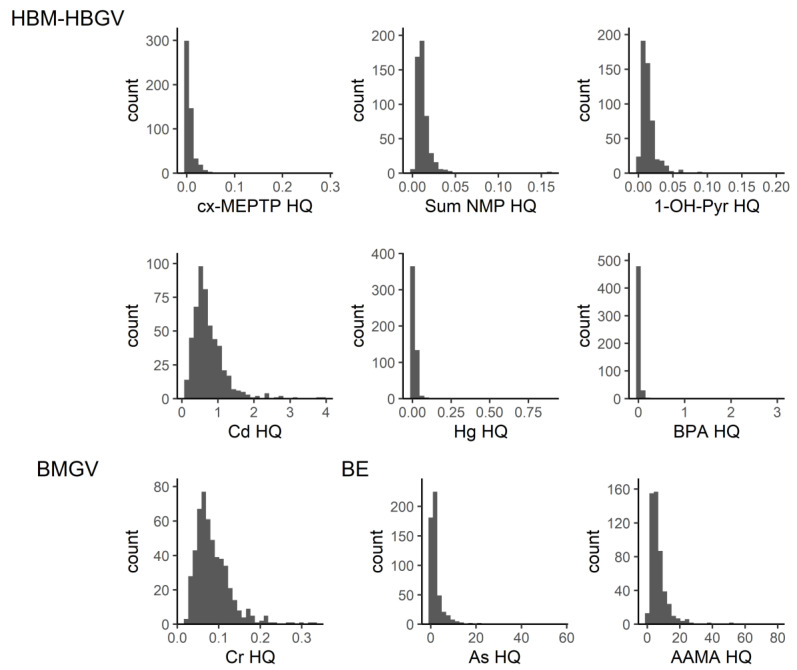
Distribution of individual hazard quotients (HQs) for substances that were not members of a community (see Figure 1) or were the only substances in that community for which a guidance value (HBM-HBGV or equivalent) was identified. The HQs for arsenic and acrylamide are based on BEs (biomonitoring equivalents), the HQ for chromium is based on a BMGV, while the HQs for cx-MEPTP (1-mono-(2-ethyl-5-carboxyl-pentyl) benzene-1,4-dicarboxylate), NMP (N-methyl-2-pyrrolidone), pyrene, cadmium, mercury and bisphenol A are based on HBM-HBGVs.

Individual HQ values for substances involved in communities I to IV are shown in Figure 4. Figure 5 depicts the individual HQ values for substances that were not part of a community or were the only substances in that community for which a guidance value (HBM-HBGV or equivalent) was identified. It can be seen from Figure 5 that AAMA (acrylamide metabolite) had the highest HQ values.

## 4. Discussion

In this study, a novel concept for using the HI methodology to identify exposure to mixtures that may be of concern from a public health perspective has been demonstrated. We first identify real-life mixtures as communities of co-occurring chemicals through a network analysis of HBM data, and then apply toxicology-based biomonitoring guidance values to gauge the hazard potential of, and thus rank, newly discovered real-life mixtures.

In the first screening, the overall HI (for the 17 substances with an available guidance value combined) exceeded the value of one in 100% of the studied population, indicating that all people had exposures of concern. It should be noted that this HI is calculated across less than half of the chemicals measured in the study participants. Rather than subsequently examining compound by compound whose biomarkers drive the HI to be above one, we chose to use the network analysis to identify mixture communities. Identifying communities of patterns of real-life co-occurring chemicals adds value by allowing prioritization and focus on those co-occurrence patterns that have the highest HIs. This is one of the first studies to derive HIs based on real-life mixture exposures across multiple chemical families. Four of the seven communities derived from the network analysis had HIs of concern based on more than two HQs; however, due to the lack of guidance values for all compounds evaluated, we do not know if there would have been more communities or compounds which could lead to higher HI or HQ values. Community VII contained acrylamide metabolites AAMA and GAMA along with metabolites from aprotic solvents and benzene. Although we could not calculate other HQs for this community due to the lack of biomonitoring guidance values, based on AAMA, this community would already be of concern. Additionally, based on our analysis, we might prioritize evaluation of exposure to phthalates as a group, rather than the individual phthalates with high HQs, as we know that these chemicals tend to occur together in the human body.

In our network analysis of GerES V, in community I, a small percentage (5.8%) of individuals had HI values greater than one, while community II had HI values well below one. The substances in these two communities were primarily phthalate metabolites. Of the metabolites, MiBP (parent compound DiBP) had an HQ greater than one in 12 out of 515 individuals, while MnBP (parent compound DnBP) had an HQ greater than one in one out of 515 individuals. In a study by Lange et al. of cumulative exposure to phthalates from a combined group of studies, including GerES V, in a similar population of children and adolescents, DnBP and DiBP contributed the most to the HIs. The geometric mean HI of the GerES V population was 0.44, with the 95th percentile HI of 1.77 [32]. This is similar to our study where the geometric mean HI was 0.36, and 95th percentile was 1.09. The Lange et al. study also concluded that over half of children and adolescents across Europe have phthalate mixture exposures which are driven by multiple compounds in the mixture, rather than single substances. Next steps aimed at reducing health risks should, in our view, focus on possible sources of (combined) exposure and exposure routes as well as opportunities for reducing exposure.

Lange et al. only evaluated phthalate mixtures. Our study also identified other communities through the network analysis, but due to the lack of established HBM-HBGVs, we could use the HI approach only for a few other communities. These include a community comprising metabolites of the phthalate substitute DINCH and a community that consists of selenium and chromium (plus antimony and the aprotic solvent metabolite HMSI). For the latter community, a small proportion of the individuals studied had an HI greater than one. Given that only two of the four substances in this community were included in the toxicity weighting, this combination of chemical substances should be further investigated. Again, better insight into possible exposure routes and sources of combined exposure will help to identify risk management options.

HBM-HBGVs (or equivalents) were available for less than half of the measured HBM levels, which leads to an underestimation of HIs of the actual communities. Missing HBGVs for members of the community present a problem and can cause the hazard to be underestimated. Of the 51 priority HBM4EU substances, 17 substances, represented by 20 biomarkers (see Table 1) had an HBGV. Of the eight communities shown in Figure 1, six had at least one HBGV, but two communities had only one HBGV, due to which, it was not possible to evaluate the mixture toxicity. Incorporating available BE values improved the coverage, but only by adding three more substances. For the large community studied in this network, about 50% of the biomarkers had an associated HBGV value. While the complete hazard indices of the communities cannot be calculated with missing values, using an incomplete set and still finding exceedance are a reason for concern and requires further investigation. HBGVs may also change as information on chemicals changes—which means that the HIs may differ depending on the HBGVs used. Incorporation of metabolites is also something to be considered, as HBGVs may not be available for all metabolites of a parent compound or may only be available for the sum of compounds.

Two individual compounds indicated high risks. These were arsenic and acrylamide. The main source of both acrylamide and arsenic for the general population is diet. Acrylamide is formed when starchy foods are cooked, particularly baked, fried, or roasted. The European Food Standards Agency’s (EFSA) risk assessment for this substance indicates that exposure could potentially raise cancer risks [33]. For arsenic, various types of food can contain arsenic. Seafood contributes greatly to exposure for certain forms of organic arsenic (arsenobetaine), but this is generally considered of less concern. A study of arsenic exposure for the German population estimated that, for infants and children, the majority of their exposure would come from the grains, especially rice-based products [34]. For adolescents, coffee, teas and infusions also contributed a large percent to exposure. High exposure to arsenic may occur in certain areas where groundwater has high arsenic content. However, this is unlikely to be influential at the population level.

Arsenic was not highly correlated with exposure to other substances, but acrylamide was in a community that included other substances, particularly benzene (metabolite SPMA), CIT/MIT (metabolite NMMA) and aprotic solvent NMP (metabolite HNMP), although biomonitoring guidance values were not identified for these. There is a possibility that the HI for this community may be even higher, with additional biomarkers contributing to the HI.

The derivation of HBM-HBGVs is an area of uncertainty. To reduce this, we prioritized guidance values issued by HBM4EU, or government regulatory agencies, because these have undergone extensive evidence review, including studies in humans and animals. However, even with values derived from human studies, two biomarkers, pyrene and chromium, were from occupational guidance values, which would likely be too high for the population in our study, which were children and adolescents. In three cases, we included biomonitoring equivalents, which are estimated using modelling approaches to estimate how maximum tolerable daily intakes would translate into biomonitoring values. BEs are not necessarily derived based on committee review, as those are set by regulatory agencies, and therefore were only used if a regulatory guideline was not available. The guidance values for arsenic and acrylamide, the two substances with HQs greater than one in much of the population, were derived from biomonitoring equivalents. However, while the BE for arsenic was based on data from studies of human populations, that of acrylamide was based on animal studies [30].

In general, the robustness of a BE depends on the quality and relevance of the exposure guidance values and pharmacokinetic data used in its derivation. In the case of arsenic, the uncertainty factors associated with the three exposure guidance values that were available for the BE calculation ranged from 3 to 10. For acrylamide, the uncertainty associated with the use of animal-based studies only would be the concern. Similarly, for cadmium and selenium, we also used BEs for the guidance values, but these were not risk drivers with substantial contributions to the HI.

One approach around the lack of HBM-HBGVs is to estimate exposure intakes from biomarker values and use reference doses (RfDs) to calculate the HQs and HI, as Kortenkamp et al. did for a variety of substances related to declining semen quality [35]. However, this presents similar issues as with BEs, as the certainty depends on the relevance of RfDs to human data and the quality of the pharmacokinetic data. For calculation of exposure HIs, the use of biomonitoring data allows us to better evaluate the actual internal exposure mixtures, which may differ from those estimated from external exposure models. Therefore, the use of a comparable reference value (i.e., one based on biomonitoring data in humans) would be preferable.

Another way in which HBM-HBGVs could be derived is through adjustment of occupational values, as there are more biological exposure indices or guidance values for occupational settings than for the general population. These could be scaled for different age groups, accounting for differences in metabolism and duration of exposure. For children, a sensitivity factor could be added due to the potential vulnerability of the developing body compared to an adult. This adjustment effort could be implemented through an official review committee, similar to those which have derived the guidance values we have used in Table 1 and referenced in Table S2.

The HI approach assumes that the cumulative effect of the doses of each mixture component is additive, and that different substances have a common mode of action with a common health outcome. However, it can be reasonably assumed that HBM-HBGVs for different chemicals are derived based on different health effects and different modes of action. Our approach does not consider any commonality in the mode of action or health outcome, and might therefore be considered relatively conservative. Thus, the results should be interpreted with caution. Should effects be common and synergistic, the hazard index would underestimate mixture toxicity, and should effects be antagonistic, the opposite would be true. Thus far, however, synergistic or antagonistic effects have been rarely reported. For mixture communities where HI exceeds one, further examination of toxic effects should be evaluated, such as considering the mode of action of each component and common health endpoints. In addition, various uncertainty factors may be applied in the derivation of guidance values, adding to the difficulty in comparing HQs. A point of departure index (PODI) approach, e.g., no or low observed adverse effect level (NOAEL/LOAEL), may provide a more robust approach to calculate HQs.

This proof-of-concept study shows that toxicity weighting can be applied at the level of identified communities, but the ability to effectively use this for chemical risk assessment is limited by the lack of HBGVs. Coupling the HI approach with a method for finding exposure-related co-occurrence patterns in population HBM data allows us to better prioritize between mixtures for risk management. An approach with wider coverage of toxicity weights is needed. The approach suggested by Zhao and co-workers [36], where they used text mining approaches, bringing together data from multiple toxicity and exposure databases, may prove fruitful for better coverage. However, the data in that database are mainly from animal experiments from a wide range of different experimental designs, tests and assays, which may make comparison to HBM values less appropriate.

This hazard index approach would be particularly beneficial in situations where there are population-level biomonitoring programs. In Kolossa-Gehring 2023, the HBM4EU coordinator lays out lessons learned from the program for the success of future biomonitoring programs. Four points are emphasized: (1) the need for a clear and well-defined consortium agreement, (2) a clear role for national government policy-makers in the program’s governance, (3) the need for a well-organized and financed scientific governance structure and (4) a clear and equal set of funding rates for all activities [37]. Future work should prioritize the generation of human-biomonitoring-based guidance values. Additionally, there needs to be consideration of how to deal with multiple metabolites, and matrices, for substance biomonitoring.

## Figures and Tables

**Table 1:** Guidance value used for the toxicity weighting.

Substance (Biomarker)	Value	Type	Ref.
BBzP (MBzP)	2000 µg/L	HBM-GV(GenPop)	[14]
DiBP (MiBP)	160 µg/L	HBM-GV(GenPop)	[14]
DnBP (MnBP)	120 µg/L	HBM-GV(GenPop)	[14]
DEHP Sum 1 (OH-MEHP + oxo-MEHP)	340 µg/L	HBM-GV(GenPop)	[14]
DEHP Sum 2 (OH-MEHP + cx-MEPP)	380 µg/L	HBM-GV(GenPop)	[14]
DINCH Sum (oxo-MINCH + cx-MINCH)	3000 µg/L	HBM-GV(GenPop)	[14]
DPHP (oxo-MPHP)	190 µg/L	HBM-GV(GenPop)	[14]
DEHTP (cx-MEPTP)	1800 µg/L	HBM-I	[18]
Mercury	7 µg/L	HBM-I	[18]
Cadmium (Cd)	0.1 µg/g creatinine	HBM-GV, 10 years and younger	[17]
Cadmium (Cd)	0.2 µg/g creatinine	HBM-GV, 11–20 years	[17]
Bisphenol A	135 µg/L	HBM-GV(GenPop)	[17]
NMP Sum (5-HNMP + 2-HMSI)	10,000 µg/L	HBM-GV(GenPop)	[17]
Pyrene (1-OH-Pyr)	4 μmol/mol creatinine	BMGV(working population)	[28]
Chromium (Cr)	10 μmol/mol creatinine	BMGV(working population)	[28]
Acrylamide (AAMA)	13 µg/L	BE	[29]
Arsenic (As total = sum of inorganic As, DMA, MMA)	6.4 µg/L	BE	[30]
Selenium (Se)	90 µg/L	BE	[31]

Abbreviations: MBzP (mono-benzyl phthalate), MiBP (mono-isobutyl phthalate), MnBP (mono-n-butyl phthalate), 5OH-MEHP (mono(2-ethyl-5-hydroxy-hexyl) phthalate), 5oxo-MEHP (mono(2-ethyl-5-oxo-hexyl) phthalate), oxo-MINCH (cyclohexane-1,2-dicarboxylate-mono-(7-oxo-4-methyl)octyl ester), cx-MINCH (cyclohexane-1,2-dicarboxylate-mono-(7-carboxylate-4-methyl)heptyl ester), oxo-MPHP (6-Oxo-Mono-propyl-heptyl phthalate), 5cx-MEPTP (1-mono-(2-ethyl-5-carboxyl-pentyl) benzene-1,4-dicarboxylate), 5-HNMP (5-hydroxy-N-methyl-2-pyrrolidone), 2-HMSI (2-hydroxy-N-methylsuccinimide).

## Data Availability

Summary data are listed in Supplementary Table S1. As the individual data are considered pseudonymized data, these cannot be made public according to the European General Data Protection Regulation.

## Supplementary Materials

The following supporting information can be downloaded at: https://www.mdpi.com/article/10.3390/toxics11050408/s1, Table S1: Descriptive statistics for substances included in the network analysis of the GerES V subsample (unweighted) from 515 participants; Table S2: Types of Human Biomonitoring Health-Based Guidance Values (HBM-HBGVs); Table S3: Substances used for toxicity weighting in network analyses.
