# Supplementary material for: Toxicity Weighting for Human Biomonitoring Mixture Risk Assessment: A Proof of Concept
Source: Toxics. 2023 Apr 26;11(5):408. doi: 10.3390/toxics11050408 (PMC10223739; doi:10.3390/toxics11050408)
Supplement: Supplementary file 1 [file toxics-11-00408-s001.zip › toxics-2262271 SM.pdf]

toxics

Toxicity Weighting for Human Biomonitoring Mixture Risk Assessment: A Proof of Concept

Miranda M. Loh [1,*], Phillipp Schmidt [2], Yvette Christopher de Vries [1], Nina Vogel [2], Marike Kolossa-Gehring [2], Jelle Vlaanderen [3], Erik Lebret [3,4] and Mirjam Luijten [5]

Table S1. Descriptive statistics for substances included in the network analysis of the GerES V subsample (unweighted) from 515 participants.

Substance Group	Substance	Biomarker	N < LOQ	% < LOQ	LOQ	P05	P10	P25	P50	P75	P90	P95	GM
Elements	Cadmium	Cd	134	26.02%	0.05	<LOQ	<LOQ	<LOQ	0.06	0.09	0.12	0.15	0.06
	Chromium	Cr	40	7.77%	0.2	<LOQ	0.2	0.26	0.34	0.49	0.62	0.77	0.36
	Mercury	Hg	26	5.05%	0.02	<LOQ	0.02	0.04	0.06	0.1	0.19	0.26	0.06
	Antimony	Sb	108	20.97%	0.04	<LOQ	<LOQ	0.03	0.05	0.07	0.1	0.13	0.05
	Selenium	Se	0	0%	0.5	15.09	16.81	21.17	27.8	37.95	47.93	57.08	28.34
	Arsenic	As	0	0%	0.1	2.45	2.93	4.35	6.89	14.17	30.5	55.21	8.42
Aprotic solvents		HNMP	0	0%	2.5	17.78	22.73	31.79	48.6	73.76	107.36	152.65	49.71
		HMSI	0	0%	2	15.6	19.62	26.81	37.15	57.12	84.41	104.4	39.32
		HESI	66	12.82%	2	<LOQ	<LOQ	2.58	4.68	10.2	39.19	70.35	5.87
Acrylamide		AAMA	0	0%	1	26.84	32.62	43.56	60.5	84.27	125.31	189.67	63.22
		GAMA	0	0%	1	5.52	6.79	8.96	12.53	17.66	24.62	28.84	12.74
Phthalate substitutes	DEHTP	OH-MEHTP	171	33.2%	0.3	<LOQ	<LOQ	<LOQ	0.41	1.13	2.59	4.23	0.48
		oxo-MEHTP	103	20%	0.2	<LOQ	<LOQ	0.19	0.46	1.06	2.28	3.83	0.47
		cx-MEPTP	0	0%	0.2	1.1	1.51	2.85	6.22	15.45	36.64	54.09	6.76
	DINCH	cx-MINCH	1	0.19%	0.05	0.21	0.29	0.49	1.02	2.11	4.91	7.8	1.08
		OH-MINCH	1	0.19%	0.05	0.41	0.54	0.98	2.13	4.66	9.48	14.72	2.22
		oxo-MINCH	8	1.55%	0.05	0.15	0.21	0.39	0.93	2.03	4.6	7.16	0.94
Phthalates	DEHP	MEHP	67	13.01%	0.5	<LOQ	<LOQ	0.71	1.22	2.04	3.35	4.19	1.2
		OH-MEHP	0	0%	0.2	3.12	3.94	5.87	8.98	13.94	21.69	28.84	9.25
		oxo-MEHP	0	0%	0.2	1.96	2.52	4.08	6.42	10.49	15.89	21.63	6.47
		cx-MEPP	0	0%	0.2	3.45	4.03	6.1	9.92	16.9	26.26	35.81	10.17
	BBzP	MBzP	2	0.39%	0.2	0.69	0.9	1.45	2.38	4.75	10.36	17.46	2.77
	DnBP	MnBP	0	0%	1	6.05	7.81	12.04	18.18	28.67	40.34	54.79	18.39
		OH-MnBP	4	0.78%	0.25	0.6	0.78	1.25	2.12	3.49	5.24	7.27	2.11
	DiBP	MiBP	0	0%	1	7.29	9.04	13.54	21.36	33.58	58.2	87.22	22.34
		OH-MiBP	0	0%	0.25	2.28	3.02	4.7	7.52	12.17	21.02	30.15	7.78
	DEP	MEP	0	0%	0.5	5.09	6.83	10.96	17.76	32.05	65.95	113.45	19.75
	DiNP	OH-MiNP	0	0%	0.2	1.88	2.28	3.35	5.27	8.73	15.12	24.62	5.76
		oxo-MiNP	0	0%	0.2	0.73	0.92	1.39	2.17	3.66	6.35	9.65	2.36
		cx-MiNP	0	0%	0.2	1.52	1.82	2.88	4.55	7.5	12.48	19.47	4.87
	DiDP	OH-MiDP	4	0.78%	0.2	0.37	0.47	0.75	1.19	2.06	3.54	5.9	1.28
		oxo-MiDP	54	10.49%	0.2	<LOQ	<LOQ	0.29	0.54	0.89	1.55	2.56	0.54
		cx-MiDP	11	2.14%	0.2	0.24	0.3	0.41	0.7	1.19	2.2	3.62	0.76
	DPHP	oxo-MPHP	184	35.73%	0.25	<LOQ	<LOQ	<LOQ	0.27	0.54	1.01	1.57	0.29
	DMP	MMP	8	1.55%	1	1.49	1.93	3.21	5.07	10.44	21.45	36	6.02
PAHs		1-OH-Nap	18	3.5%	0.05	0.11	0.19	0.36	0.68	1.41	3.42	4.88	0.7

		2-OH-Nap	1	0.19%	0.05	0.92	1.15	1.86	3.15	5.89	11.06	15.89	3.38
		2-OH-Flu	54	10.49%	0.05	<LOQ	<LOQ	0.23	0.43	0.69	1.27	2.19	0.36
		1-OH-Phe	0	0%	0.005	0.04	0.05	0.08	0.12	0.2	0.34	0.46	0.13
		2-OH-Phe	4	0.78%	0.005	0.03	0.03	0.05	0.07	0.11	0.18	0.28	0.08
		3-OH-Phe	0	0%	0.005	0.05	0.05	0.08	0.11	0.18	0.3	0.4	0.12
		4-OH-Phe	2	0.39%	0.001	0.01	0.01	0.02	0.04	0.08	0.18	0.26	0.04
		9-OH-Phe	14	2.72%	0.005	0.01	0.02	0.03	0.05	0.09	0.19	0.28	0.05
		1-OH-Pyr	7	1.36%	0.01	0.03	0.04	0.06	0.09	0.14	0.22	0.29	0.09
Parabens	Methylparaben	MeP	13	2.52%	0.5	0.8	1.04	1.9	4.37	19.61	122.99	321.93	7.02
	Ethylparaben	EP	164	31.84%	0.5	<LOQ	<LOQ	<LOQ	0.62	1.42	4.38	10.39	0.72
Bisphenols	Bisphenol A	BPA	19	3.69%	0.5	0.52	0.67	1.03	1.6	2.88	4.8	6.91	1.77
Other	Lysmeral	TBBA	0	0%	0.2	2.12	2.87	4.49	8.16	15.45	24.19	35.56	8.49
	CIT/MIT	NMMA	0	0%	0.5	2.48	2.99	3.92	5.31	7.53	10.24	12.2	5.47
	Butylhydroxytoluol	BHT	1	0.19%	0.2	0.58	0.73	1.23	1.98	3.45	6.22	9.52	2.1
	Benzene	SPMA	12	2.33%	0.02	0.02	0.03	0.05	0.08	0.14	0.26	0.44	0.09

Table S2. Types of Human Biomonitoring Health-Based Guidance Values (HBM-HBGVs).

**HBM-HBGVs (Acronym) Country**	**Description**
Environmental:	
Human biomonitoring guidance value for the general population ($HBM\text{-}GV_{GenPop}$)<br><br>Germany	The HBM-GV(GenPop) represents the concentration of a substance or its specific metabolite(s) in human biological media (e.g., urine, blood, hair) at and below which, according to current knowledge, there is no risk of health impairment and, consequently, no need for action. They are equivalent to the HBM-I values from the German Human Biomonitoring Commission. They are derived within HBM4EU for priority substances. (Apel et al. 2020)
Human Biomonitoring I (HBM-I)<br><br>Germany	The HBM-I value derived on the basis of toxicological and epidemiological studies, represents the concentration of a substance in human biological material below which—according to the knowledge and judgement of the HBM Commission—there is no risk for adverse health effects and, consequently, no need for action. (German Human Biomonitoring Commission)
Human Biomonitoring II (HBM-II)<br><br>Germany	The HBM-II-value derived on the basis of toxicological and epidemiological studies, represents the concentration of a substance in a human biological material above which—according to the knowledge and judgement of the HBM Commission—there is an increased risk for adverse health effects and, consequently, an acute need for exposure reduction measures and the provision of biomedical advice. The HBM-II-value should thus be regarded as an intervention or action level. (German Human Biomonitoring Commission)
Biomonitoring equivalent (Be)<br><br>-	The concentration or range of concentrations of a chemical or its metabolite in a biological medium (blood, urine or other medium) that is consistent with an existing health-based exposure guideline. This could be a non-cancer health-based guidance value such as a reference dose (RfD) or tolerable or acceptable daily intake (TDI or ADI) or a cancer-based exposure guidance value such as a risk-specific dose (e.g., the dose associated with a 1 × 10^{-4} cancer risk). (Hays and Aylward, 2012).
Occupational:	

Human biomonitoring guidance value worker (HBM-GV$_{Worker}$)<br><br>-	The HBM-GV(Worker) represents the concentration of a substance or its relevant metabolite(s) in human biological media aiming to protect workers exposed to the corresponding substance regularly (each work day) and over the course of a working life from the adverse effects related to medium- and long-term exposure. They are derived within HBM4EU for priority substances. (Apel et al. 2020).
Biologische Arbeitsstoff-Toleranzwerte (Biological Tolerance Value) (BAT)<br><br>Germany	The German Commission establishes BAT ("Biologische Arbeitsstoff-Toleranzwerte": biological tolerance values) and BLW ("Biologische Leit-Werte") values to enable the evaluation of the risk to an individual's health which results from exposure to a substance at the workplace.<br><br>The BAT value describes the occupational-medical and toxicologically derived concentration for a substance, its metabolites or an effect parameter in the corresponding biological material, at which the health of an employee generally is not adversely affected, even when the person is repeatedly exposed during long periods. BAT values are based on a relationship between external and systemic exposure or between the systemic exposure and the resulting effect of the substance. The derivation of the BAT value is based on the average of systemic exposures. (DFG 2020)
Biological Exposure Index (BEI)<br><br>New Zealand	The BEI is a limit value that represents the level of exposure the typical worker can experience without adverse health effects. The values are established by the American Conference of Governmental Industrial Hygienists (ACGIH). (New Zealand Government 2020)
Biological Limit Values (BLVs)<br><br>France	The biological limit values are recommended by ANSES as biological exposure markers which are considered to be relevant in the workplace. They are intended to protect workers from harmful effects related to exposure to the chemical in question over a medium- or long-term. They take into account repeated exposure throughout a worker's working life.<br><br>Depending on the available data, the recommended biological limit values do not all have the same meaning:<br>- if the body of scientific evidence is sufficient to quantify a dose/response relationship with certainty, the biological limit values (BLVs) will be established on the basis of health data (no effect for threshold substances or risk levels for non-threshold carcinogens);<br>- in the absence of such data for substances with threshold effects, BLVs are calculated on the basis of the expected concentration of the biomarker of exposure (BME) when the worker is exposed to the 8 h OEL.<br>For carcinogens, in the absence of sufficient quantitative data, the biological limit values are calculated on the basis of another effect (pragmatic BLVs).<br><br>These last values do not guarantee the absence of health effects, but aim to limit the exposure to these substances in the workplace. (ANSES 2013, ANSES 2015)
Biologische Leit-Werte (BLW)<br><br>Germany	The German Commission establishes BAT ("Biologische Arbeitsstoff-Toleranzwerte": biological tolerance values) and BLW ("Biologische Leit-Werte") values to enable the evaluation of the risk to an individual's health which results from exposure to a substance at the workplace.<br><br>The BLW ("Biological guidance value") is the amount of a chemical substance or its metabolites or the deviation from the norm of biological

	parameters induced by the substance in exposed humans, which serves as an indicator for necessary protective measures. BLWs are assigned only for hazardous materials, for which the available toxicological or occupational-medical data are insufficient for the establishment of BAT values (i. e. for carcinogenic substances and suspected carcinogens in categories 1 to 3 and for non-carcinogens, for which the toxicological data are inadequate).<br><br>BLW values are generally established on the assumption that persons are exposed at work for at most 8 h daily and 40 h weekly during their working lives).<br>The BLW is based on occupational-medical information as to the effects of handling the hazardous material together with toxicological data. (DFG, 2020)
Biological Monitoring Guidance Values (BMGVs)<br><br>United Kingdom	These have been established by the Health and Safety Executive UK. BMGVs are either based on a relationship between biological concentrations and health effects, between biological concentrations and exposure at the level of the Workplace Exposure Limits or on data collected from a representative sample of workplaces correctly applying the principles of good occupational hygiene practice.<br>The types of data that are available will vary between substances, and therefore the route taken to derive the BMGV will vary between substances. (HSE UK, 2020)

Table S3. Substances used for toxicity weighting in network analyses.

HBM4EU_Toxicity-weighing_Supplementary material_Table_S3.xlsx
